# Impact of Multimodal Rehabilitation Protocol in a 20-Year-Old Patient With Cherubism Undergone Facial Surgery: A Rare Case Report

**DOI:** 10.7759/cureus.56191

**Published:** 2024-03-14

**Authors:** Shifa S Sheikh, Vrushali Athawale, Tejaswini Fating

**Affiliations:** 1 Community Health Physiotherapy, Ravi Nair Physiotherapy College, Datta Meghe Institute of Higher Education and Research, Wardha, IND; 2 Community Health Physiotherapy, Ravi Nair Physiotherapy College, Datta Meghe Institute of Medical Sciences, Wardha, IND

**Keywords:** cherubism, hospital anxiety and depression scale (hads), breathing exercises, facial pnf, rehabilitation, management of cherubism

## Abstract

Cherubism, a rare autosomal dominant disorder, presents with symmetrical, painless jaw extension due to fibrous tissue ossification, often referred to as hereditary fibrous dysplasia of the jaw. It typically manifests with progressive mandibular and maxillary swelling from childhood to adolescence, with exacerbation over time. A 20-year-old male presented with facial and jaw swelling, causing restricted jaw movements. Computed tomography confirmed the cherubism diagnosis. Subsequently, the patient underwent oral surgery for bone shaving and shaping. Post-surgery, a five-week physiotherapy regimen was initiated, emphasizing joint mobility preservation through active range-of-motion exercises and proprioceptive neuromuscular facilitation for facial expression and dyspnea alleviation. Following physiotherapy, significant improvements were observed, including enhanced respiratory function, increased cervical muscle strength, improved respiratory clearance, and reduced anxiety and depression levels. This case highlights the importance of physiotherapy in cherubism rehabilitation, a novel approach deserving further exploration.

## Introduction

A rare condition known as cherubism is an inherited autosomal dominant disorder characterized by symmetrical, painless expansion of the jaws caused by the replacement of bone with fibrous tissue [1]. However, the condition is also known as hereditary fibrous dysplasia of the jaws [1]. Only around 300 cases of cherubism are documented in the literature, indicating its extreme rarity. Estimating the frequency of this disorder is difficult due to its scarcity. Cherubism affects both males and females equally, with cases reported across diverse racial and ethnic backgrounds [2]. There are two types of cherubism: nonhereditary (nonfamilial) and hereditary (familial) [3]. Bilaterally symmetrically enlarged cheeks, especially at the mandibular angles, and an upward tilting of the eyes are the characteristics of cherubism. The mandible and maxilla first protrude in early childhood and then progressively worse until adolescence. However, pathological situations may cause these structures to deteriorate and cause functional, aesthetic, and psychological damage, which ultimately affects quality of life [4].

The primary factor contributing to the pathophysiology of cherubism is mutations in the SH3 domain-binding protein 2 (SH3BP2) gene, situated on chromosome band 16.3 on the short arm of chromosome 4 (4p16.3) [5]. Usually, these mutations are inherited autosomal dominant, meaning that a heterozygous individual having one copy of the mutant gene will display the cherubism-defining traits [6]. The protein SH3BP2 participates in signaling networks that control cell proliferation, differentiation, and bone remodeling, leading to aberrant activation. Overactivation of the osteoclast is caused by aberrant signaling resulting from a mutation in SH3BP2 [7]. The body's reaction to the osteoclastic activity produces new bone creation, but the aberrant activation of the osteoclast leads to the breakdown of bone. However, this chaotic process results in fibrous tissue and cyst-like lesions that are typical of cherubism [8]. In addition to facial deformities, problems could include upper airway blockage, sleep apnea, optic neuropathy, vision loss, language impairments, and trouble eating, chewing, and swallowing due to altered tooth development and appearance [9].

The management of cherubism encompasses a range of surgical procedures tailored to address the specific manifestations of the condition. These procedures aim to alleviate symptoms and improve functional and aesthetic outcomes. Common surgical interventions include bone shaving and shaping, which entail the precise removal of excess bone from the jaw to reduce swelling and restore facial symmetry. Orthognathic surgery is utilized to correct bite misalignments and enhance facial aesthetics by repositioning the upper or lower jaw [10]. Orthodontic procedures, such as braces or dental appliances, are employed to align teeth and optimize jaw function. In cases of significant bone loss, bone grafting may be performed to replace missing bone tissue and enhance jaw structure. Additionally, reconstructive surgery may be necessary in severe cases to rebuild jaw and facial structures [11]. Despite the benefits of surgical intervention, patients may experience postoperative complications such as swelling, pain, scarring, stiffness, and muscle weakness. These complications, if not managed effectively, can potentially lead to cervical contractures and other long-term sequelae. Therefore, meticulous postoperative care and rehabilitation are essential to optimize patient outcomes and minimize complications. The physiotherapeutic approach focuses on facial muscle rehabilitation, restoring range of motion, preventing stiffness, improving functional ability, and enhancing overall recovery [10]. While surgical approaches have been extensively studied, limited literature exists on the utilization of physiotherapy in cherubism cases. This case report aims to fill this gap by exploring the potential benefits of physiotherapy in improving postoperative outcomes and enhancing patient recovery in cherubism. By emphasizing the physiological effects of physiotherapy in similar conditions and its potential impact on cherubism management, this study seeks to draw attention to this novel perspective in treating such conditions.

## Case presentation

Patient information

The patient is a 20-year-old male from the age of 13, he began to notice swelling on both sides of his face and jaw, which progressively worsened over the years. The timeline of the onset of symptoms, progression, and computed tomography (CT) findings is given in Table 1. The patient received homeopathic treatment five years ago. He gave a history of betel nut chewing occasionally for two to three years. The patient gave no significant family history of cherubism either from paternal or maternal relatives. The patient visited multiple hospitals for treatment and visited private hospital. Due to a lack of significant treatment, the patient visited Acharya Vinobha Bhave Rural Hospital on October 13, 2023, with a chief complaint of swelling over the bilateral jaw and face and difficulty in facial movement and eating. The patient had complaints of pain in the mental region, which was intermittent, dull aching, and non-radiating in nature. Due to the pain patient faced difficulty in chewing and swallowing causing malnourishment, and facial deviation resulting in facial disfigurement. Following physical examination CT scan was done showing expansion of bony calvarium with an intact cortex and loss of trabeculae, giving homogenous ground glass appearance noted in the mandible and involving the bilateral floor of orbits, bilateral maxilla obscuration of sinuses. Pterygoid plates and maxillary sinuses with partial obscuration of sinuses. After a thorough examination, the oralmaxillofacial surgeon suggested bone shaving and shaping, and the patient was admitted to the oral surgery ward. On October 25, 2023, bone shaving and shaping were done under general anesthesia, and the patient was shifted to the intensive care unit (ICU) for two days and then was shifted to the ward. The patient was referred for physiotherapy postoperatively for further management.

**Table 1:** Timeline of symptoms onset, progression and CT scan findings. 3D: 3 dimensional, CT: computerized tomography

Age of symptoms onset	Symptom progression	Year of symptoms occurred and investigations done	3D CT scan report findings
13 years of age	swelling over the bilateral sides of the face and jaw, which increased gradually over time	2016	No investigations were done.
15 years of age	tooth exfoliation of the lower anterior part	2018	Multiloculated cystic masses with symmetric involvement of mandible and maxilla, it was associated with diffuse enlargement of bilateral mandible and maxilla with widening of diploic space.
17-18 years of age	blurred vision from the left eye	2021	Multiple lucent expansile lesions within the maxilla and mandible, with the soap-bubble appearance.
18-19 years of age	nerve paraesthesia over the lateral to the right ala of the nose	2022	No investigations were done.
20 years of age	swelling over bilateral jaw and face and difficulty in facial movement and difficulty in chewing and swallowing causing malnourishment, facial deviation resulting in facial disfigurement	2023	expansion of bony calvarium with an intact cortex and loss of trabeculae, giving homogenous ground glass appearance noted in the mandible and involving the bilateral floor of orbits, bilateral maxilla obscuration of sinuses. Pterygoid plates & maxillary sinuses with partial obscuration of sinuses.

Diagnostic assessment

**Figure 1:**
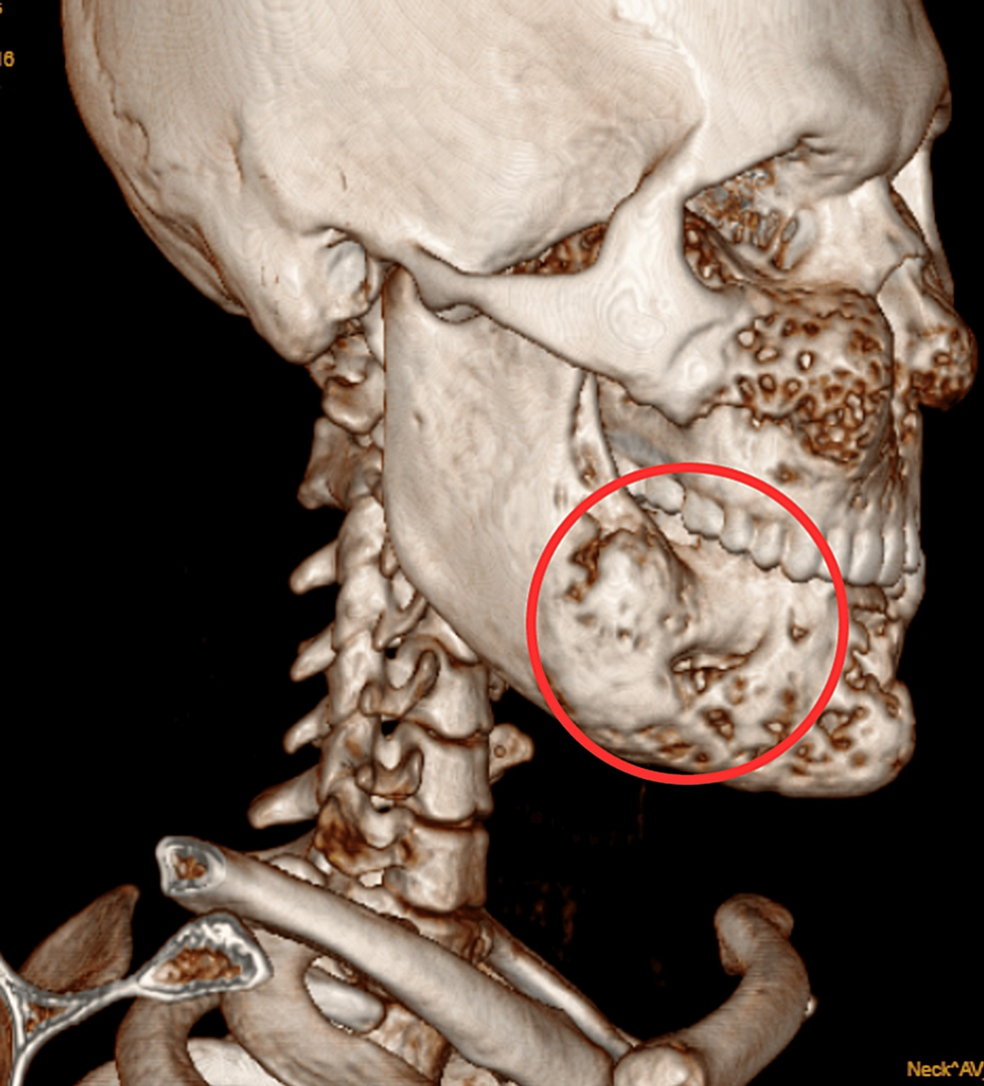
Patients computed tomography (CT) scan Red circle denotes the expansion of the bony calvarium of mandibular body.

CT Brain plain revealed expansion of bony calvarium with an intact cortex and loss of trabeculae, giving homogenous ground glass appearance noted in the mandible and involving the bilateral floor of orbits, bilateral maxilla obscuration of sinuses. Pterygoid plates and maxillary sinuses with partial obscuration of sinuses. These findings were suggestive of cherubism as shown in Figure 1.

Clinical findings

Informed consent was taken before the examination. The patient was conscious, cooperative, and oriented to time, place, and person and had an ectomorphic build. The patient had a body mass index (BMI) of 15.85 (Underweight). Pre-operatively the patient had complaints of pain in the mental region, which was intermittent, dull aching, and non-radiating in nature. On the numerical pain rating scale (NPRS), 3/10 on rest and 5/10 on jaw movement. The patient had blurry vision in the left eye.

Postoperatively on observation, the patient was seen in a long sitting position. On examination, the patient was afebrile, with a pulse rate of 78 beats per minute and a respiratory rate of 20 breaths per minute. Patient-rated pain at the suture site was sudden in onset, dull-aching, and non-radiating in nature and on the NPRS, 8/10 on mouth movements and 5/10 on rest. Postoperatively the patient's face was grossly asymmetrical due to the presence of diffused edema over the bilateral cheek region and the bilateral mandibular region. The suture length intraorally was approximately 5 centimeters. Tenderness of grade 3 was present. 

On respiratory examination, chest expansion over the second intercoastal rib and the xiphoid process was 1.5 cm and effective airway clearance was difficult. On auscultation, bilateral air entry was reduced in the lower zones of the lungs. The patient experienced dyspnea of grade 3 (stops for breath after walking 100 yards (91 m) or after a few minutes), according to the Modified Medical Research Council (mMRC). The range of motion (ROM) of the cervical joint was reduced postoperatively and the increase in ranges post-interventions is shown in Table 2. ROM was measured using a goniometer for cervical joint ranges. The muscle strength of both upper and lower limbs postoperatively was assessed as 4/5. All sensations and reflexes were intact.

**Table 2:** Range of motion (ROM) of cervical joint POD: Postoperative day

ROM of cervical joint	Pre-intervention (POD 5)	Post-intervention
Flexion	0-35^o^	0-42^ o^
Extension	0-30^ o^	0-40^ o^
Lateral flexion (right)	0-35^ o^	0-40^ o^
Lateral flexion (left)	0-35^ o^	0-40^ o^

Therapeutic intervention

**Figure 2:**
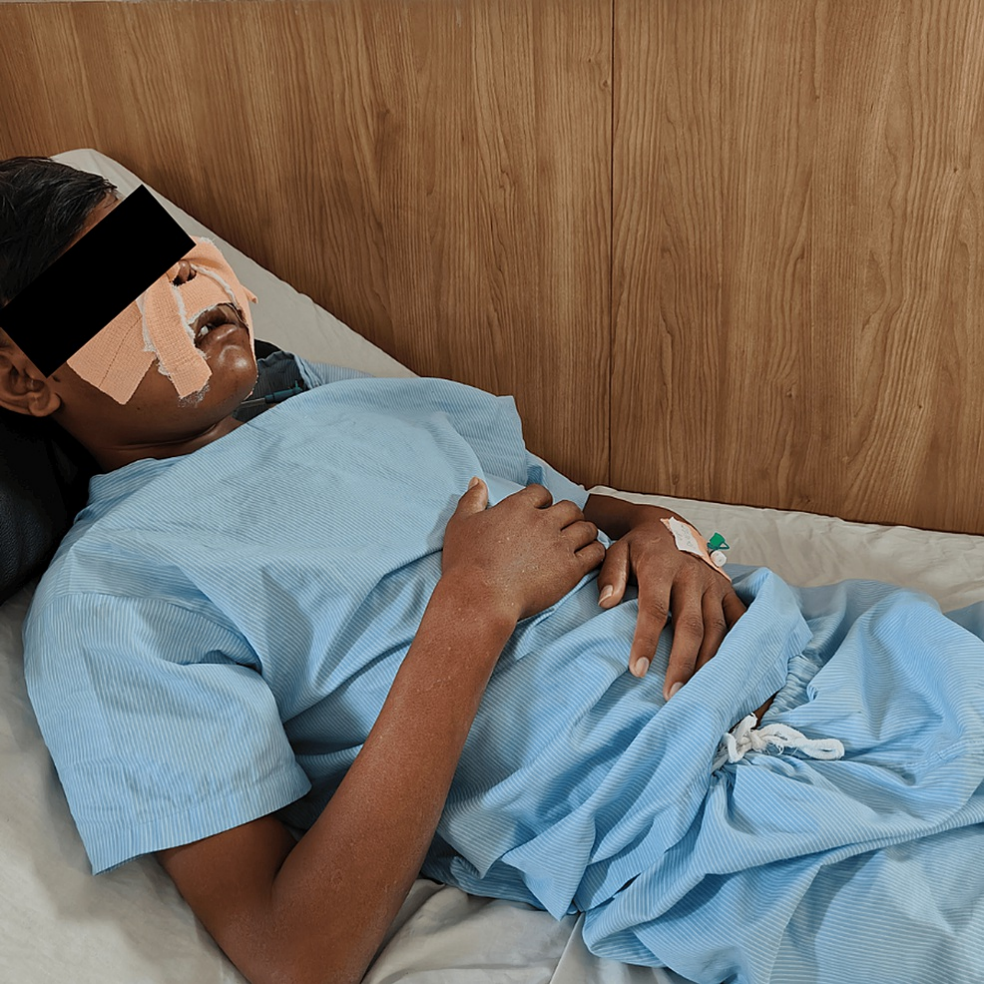
Physiotherapy intervention: Patient performing diaphragmatic breathing exercise

**Figure 3:**
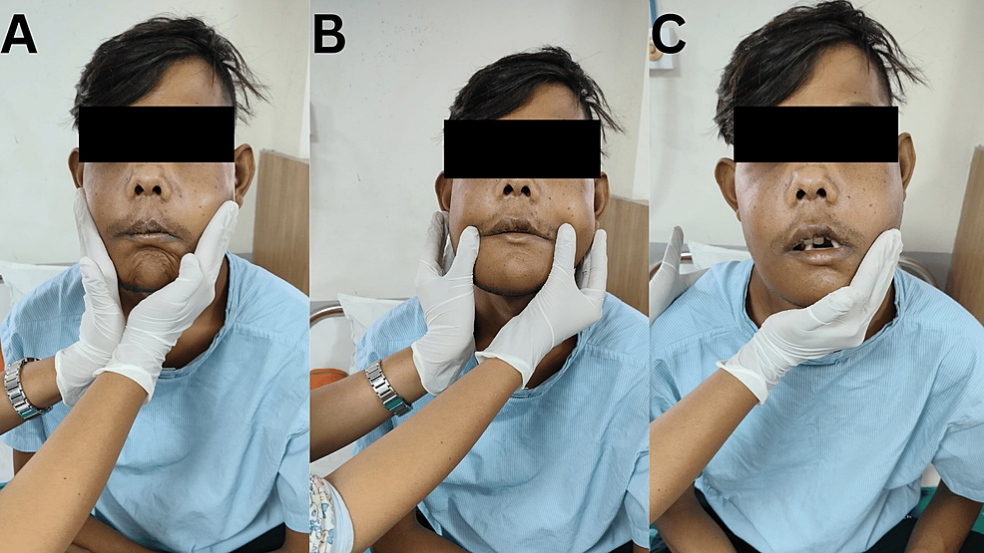
Facial Proprioceptive neuromuscular facilitation (PNF) exercises postoperatively (A) PNF for buccinator, (B) PNF for orbicularis oris, (C) PNF for masseter

The patient was taught dyspnea, revealing positions, and active ROM exercises in the hospital setup to maintain joint mobility and integrity. Table 3 provides the physical therapy protocol. Figure 2 shows the patient performing breathing exercises. Figure 3A shows the patient performing proprioceptive neuromuscular facilitation (PNF) for the buccinator muscle, Figure 3B shows the patient performing PNF for orbicularis oris, and Figure 3C shows the patient performing PNF for the masseter muscle.

**Table 3:** Physiotherapy week wise plan of care N/A: not applicable, UL: Upper limb, LL: Lower limb, ROM: Range of motion, ADL’s: Activities of daily living, kg: Kilogram, ACBT: Active cycle of breathing technique, PNF: Proprioceptive neuromuscular facilitation.

Week	Goals	Problems identified	Physical therapy protocol	dosage	rationale
Week 1- week 2	Patient Education	Lack of awareness about physiotherapy	Education and counselling of patients about the effects of exercise, effect on patient recovery, improvement in the facial movement and oropharyngeal activity.	N/A	To encourage the patient to do daily active exercises, encourage the family to do regular activities.
	Relieve dyspnoea	Difficulty in breathing due to post-operative oedema	Dyspnoea relieving positions (forward lean sitting on table with and without pillow support, high side-lying position, standing erect with hands hang slightly forward to the body)	10 times, 1 set each session and as per the dyspnoea occurs	Dyspnoea-reliving positions use pressure from the abdominal content to dome the diaphragm muscles, resulting in the facilitation of breathing.
	Improve ventilation and prevent excessive work of breathing	Due to facial swelling work of breathing has increased	Diaphragmatic breathing exercise [12] with patient in semi-fowlers position with one hand over thorax and other over abdomen to feel the abdominal rise during inspiration, glossopharyngeal breathing [13] exercise with patient in sitting position and hands supported over the thigh.	10 times, 1 set x BD x progressively increasing 5 repetitions till week 4	Glossopharyngeal breathing exercise involves galloping of air in through the mouth and throat to inhale air into the lungs; this mechanism creates negative pressure through coordinated activation of facial muscles and draws air into the lungs.
	Improve chest expansion	Reduced air entry into the lungs	Thoracic expansion with upper limb mobility in high sitting position (bed side sitting)	10 times, 1 set x BD x progressively increasing 5 repetitions till week 4	The thoracic expansion improves the air entry into the lungs and enhances the expansion of the lungs.
	Promote active expectoration of sputum	Due to muscle weakness and pain at the suture site	ACBT [14] technique involves breathing control, thoracic expansion followed by breathing control and huffing exercise all performed in sitting position.	5 cycles x 1 set x BD x since day 3 and 4. progressively increasing 5 cycles as pain subsided till the end of week 2	ACBT opens the inter-bronchiolar channel of Martins, the bronchiolar-alveolar track of Lambert, causing accumulation in the thick sputum. Active huffing improves the active expulsion of sputum.
	To reduce anxiety and depression	Appearance and swelling of the face cause anxiety in the patient.	Buteyko breathing exercise (following a relaxed exhalation, pinch the nose with index and thumb till the urge to breathe occurs), Progressive muscle relaxation.	10 times, 1 set x BD x progressively increasing 5 repetitions till week 4	Helps relax patient
Week 3-4	Improve facial movements of muscles (buccinator, orbicularis oris, nasalis, corrugator frontalis, corrugator supercilia orbicularis oculi, Mentalis)	Difficulty in pouting, clenching of teeth, blowing cheeks, smiling, lower lip protrusion)	Facial PNF [15] with Active assisted mouth movement.	10 times, 1 set x BD x progressively increasing the 5 repetitions from week 3	Improves the facial expressions.
	Improve the strength of facial muscles	Weakness of facial muscles due to incision	Strengthening of buccinator muscle, orbicularis oris (paper picking with straw)	10 times, 1 set x BD x progressively increasing 5 repetitions weekly	Strengthening of the facial muscles improves the work of chewing and speaking.
	Improve eye closure	Weakness of eye musculature	Active assisted eye-closing exercise	10 times, 1 set x BD x progressively increasing 5 repetitions weekly	Strengthens eyelid muscles and promotes
Week 3- 5	Improve cervical range of motion	Restriction in cervical ROM caused due to pain and swelling	ROM exercises of the cervical joint	10 times, 1 set x BD x progressively increasing 5 repetitions weekly	ROM exercises improve the cervical movement and improve ADL's
	Improve the strength of cervical muscles and improve swallowing	Prevent muscle weakness	Shakers exercises (Shaker exercises involve lifting the head while lying on one's back), Cervical muscle strengthening (neck isometrics, chin tuck, shoulder shrugs, scapular retractions)	10 times, 1 set x BD x progressively increasing 5 repetitions weekly	Strengthening causes proper facial contouring and preventing sagging or drooping of the soft tissue, hence enhance facial symmetry, improves oral function.
	Improve the strength of UL and LL	Maintain the UL and UL strength	Ankle pumps, Heel slides, using 1 kg weight cuffs and TheraBand for strengthening.	10 times, 1 set x BD x progressively increasing 5 repetitions weekly	Stronger upper limbs reduce the risk of falls, especially if there are alterations in facial structure affecting weight distribution. Additionally, enhancing lower limb strength improves physical function, balance, and stability.

Follow-up and outcome measures

The patient achieved notable functional milestones during the rehabilitation process, including a steadily increasing TMJ range of motion, improved functional capacity, and reduced pain. Additionally, there was a significant improvement in facial expressions and everyday activities following physical therapy sessions. Specifically, the patient demonstrated enhanced ventilation and regained the ability to perform various facial movements such as smiling, pouting, clenching of teeth, and lower lip protrusion. These improvements were indicative of enhanced respiratory function and improved neuromuscular control of facial muscles. There was effective removal of secretions.

There was a decrease in dyspnea from grade 3 to grade 1 on the mMRC scale; the strength of cervical muscles increased with a grade of 4 to grade 5. The patient’s anxiety and depression reduced (from anxiety-9 and depression-7 to anxiety 4 and depression 5) according to the hospital anxiety and depression scale [16]. The pre-intervention strength of both upper and lower limb muscles was graded as 4/5, while post-intervention strength improved to 5/5 for both limbs. According to the neck disability index, initially, the patient had a score of 50% (moderate), and the rehabilitation patient had a score of 10% (no disability). For the assessment of cervical flexors, the patient assumes a supine lying position with the back stabilized by the plinth. The patient was instructed to perform neck flexion, bringing the chin towards the chest. To evaluate cervical extensors, the patient is positioned in a prone lying position. The patient is directed to extend the neck backward against resistance. In the assessment of cervical lateral flexors, the patient is placed in a supine lying position. They are then instructed to laterally flex the neck. The manual muscle testing (MMT) of cervical muscles is shown in Table 4. Functional Independence Measure pre-intervention post-surgery was at level 5 (supervision), and post-intervention was at level 7 (complete independence).

**Table 4:** Manual muscle testing (MMT) of cervical muscles POD: postoperative day

MMT	Pre-intervention (POD 5)	Post-intervention
Cervical flexors	4/5	5/5
Cervical extensors	4/5	5/5
Cervical lateral flexors	4/5	5/5

## Discussion

Postoperative rehabilitation plays a pivotal role in cherubism management, aiming to optimize patient health and well-being following surgical intervention. This case report provides a detailed account of the rehabilitation strategies employed for a specific postoperative cherubism patient. Understanding the challenges associated with cherubism and the importance of comprehensive rehabilitation is fundamental for improving patient outcomes. The utilization of diaphragmatic breathing exercises emerges as a cornerstone of postoperative rehabilitation, with demonstrated benefits for respiratory health, stress reduction, and overall mental and physical well-being. These exercises enhance diaphragmatic function, improve lung capacity, and promote oxygenation of tissues. Additionally, manual support to the diaphragm fosters relaxation and tension reduction [12].

Glossopharyngeal breathing (GPB) presents another valuable breathing technique, particularly beneficial for individuals with respiratory disorders such as spinal cord injuries and neuromuscular conditions. GPB involves the activation of mouth and throat muscles to facilitate inhalation, thereby enhancing breathing capacity by creating a negative pressure within the oral cavity. The study reveals that GPB significantly improves cough function in patients with chronic poliomyelitis and impaired respiratory function. GPB enhances the peak rate of expiratory flow, facilitating better clearance of airway secretions. It is most beneficial for patients with very low vital capacities and high vital capacities. However, GPB does not enable patients to achieve a completely normal cough. This study focuses on evaluating enhancements in cough function, peak rate of expiratory flow, and the clearance of airway secretions. In contrast, our study assesses outcomes related to facial function, psychosocial well-being, and quality of life. Furthermore, we observed the effectiveness of GPB in our patients in promoting swallowing and facilitating airway clearance [13].

Aranha et al. highlight the efficacy of facial PNF treatment in children with Bell's palsy compared to conventional therapy methods. This approach utilizes irradiation principles to stimulate weakened facial muscles, with proactive patient involvement contributing to recovery. PNF exercises and facial massage effectively improved facial symmetry and reduced synkinesis, highlighting the potential of non-invasive rehabilitation methods. We employed the PNF technique in patients with cherubism and observed positive outcomes in facial expression [17]. By employing diagonal stretching patterns to elicit significant muscle contraction, PNF facilitates facial symmetry improvement and reduces impairment. Furthermore, repeated use of neural pathways lowers synaptic thresholds, enhancing neuromuscular responses [18].

Myofascial relaxation, home exercises, and progressive muscle relaxation techniques have also been shown to alleviate temporomandibular joint (TMJ) pain, reduce jaw movement restriction, and address bruxism symptoms, thereby improving overall well-being [17, 19]. Shakers exercises, as demonstrated by Balou et al., play a significant role in improving swallowing function through the enhancement of suprahyoid muscle strength, coordination, and endurance. In their research, it was discovered that typical adults diagnosed with dysphagia through radiological examination were administered a protocol once weekly, incorporating the use of shakers alongside various exercises. In contrast, our study solely employed shakers twice daily, revealing a notable reduction in swallowing difficulty [20].

Moreover, Craciun et al. identified a correlation between temporomandibular and cervical dysfunction with functional limitations, underscoring the importance of lower facial exercises and resistance training in enhancing muscle strength and endurance. They utilized the Neck Disability Index and observed a significant decrease in patients with temporomandibular dysfunction after three months of treatment in the experimental group, which received physiotherapy alongside conservative management. Our findings align with theirs, indicating that physiotherapy helps maintain the functional state in Cherubism patients, as evidenced by the Neck Disability Index results [21]. Resistance exercises targeting key neck muscles such as the levator scapulae, trapezius, and sternocleidomastoid muscles contribute to post-traumatic muscle healing and improved neuromuscular control, ultimately reducing the risk of injury. Additionally, research suggests that core strengthening and targeted muscle strengthening can help alleviate pain. Furthermore, we have also discovered a positive effect of strengthening exercises in patients with cherubism in reducing pain [22].

The physical therapy protocol spans several weeks, with distinct goals and interventions tailored to each stage of recovery. In the initial two weeks, the focus is on patient education, relieving dyspnea, improving ventilation, chest expansion, promoting expectoration, and reducing anxiety and depression. Daily sessions involve various exercises such as dyspnea-relieving positions, breathing exercises, thoracic expansion, and relaxation techniques, with each set comprising 10 repetitions twice a day, progressively increasing by five repetitions. In weeks three to four, the emphasis shifts towards improving facial movements and strength, as well as eye closure, with sessions including facial PNF, muscle strengthening exercises, and eye-closing exercises, each set being performed 10 times twice a day, with repetitions increasing by five weekly. Finally, in weeks three to five, the protocol targets enhancing cervical range of motion, muscle strength, stability, and strength of upper and lower limbs through a range of motion exercises, muscle strengthening, and limb-specific exercises, with frequency and intensity progressively increasing to optimize recovery and functional outcomes. Each session typically involves performing exercises 10 times per set, twice daily, with repetitions progressively increasing as the patient's tolerance and recovery allow.

In summary, the rehabilitation strategies outlined in this case report offer a comprehensive approach to postoperative care for patients with cherubism. Drawing from previous studies and clinical experiences, our therapeutic interventions encompassed a range of techniques aimed at enhancing respiratory function, promoting facial muscle strength and mobility, and improving overall well-being. The comparison with existing literature suggests that our approach, particularly the incorporation of diaphragmatic breathing exercises and facial PNF techniques, yielded promising outcomes in terms of reducing dyspnea, enhancing facial expressions, and improving cervical range of motion. The future perspective of the study could involve longitudinal follow-up studies to assess the long-term effects of the rehabilitation protocol on facial function and quality of life. Comparative research could evaluate its efficacy against alternative approaches, while qualitative methods could explore patient experiences. Overall, the aim is to optimize rehabilitation strategies for individuals with cherubism undergoing facial surgery, with a focus on enhancing functional outcomes and well-being. Our case report underscores the importance of postoperative rehabilitation in cherubism.

## Conclusions

The post-surgical rehabilitation approach is effective, resulting in significant improvements in both well-being and physical functionality. This case study mentioned above offers a thorough treatment plan for people with cherubism who have undergone facial surgery. Although the patient's full recovery was not achieved during treatment, most therapeutic goals were acquired, including enhanced muscle strength, a steadily increasing TMJ range of motion, increased functional capacity, pain reduction, and improved facial expressions and everyday activities following physical therapy sessions. The patient’s ventilation increased, and the patient was able to do facial movements like smiling, pouting, clenching of teeth, and lower lip protrusion.
